# Optimal Tryptophan Improved Growth and Regulated Agonistic Behavior of Oriental River Prawn, *Macrobrachium nipponense*

**DOI:** 10.1155/anu/4002048

**Published:** 2025-10-09

**Authors:** Shiqian Cao, Qian Chen, Qianhui Wang, Bo Liu, Xiaochuan Zheng, Qun-Lan Zhou

**Affiliations:** ^1^Wuxi Fisheries College, Nanjing Agricultural University, No. 69 Xuejiali, Wuxi 214128, China; ^2^School of Fisheries and Life Science, Dalian Ocean University, Dalian 116023, China; ^3^Freshwater Fisheries Research Center, Chinese Academy of Fishery Sciences, No. 9 Shanshui East Road, Wuxi 214081, China

**Keywords:** 5-hydroxytryptamine, agonistic behavior, growth performance, *Macrobrachium nipponense*, protein and lipid metabolism, tryptophan

## Abstract

Tryptophan, recognized as the third limiting amino acid, plays a crucial physiological function. The optimal tryptophan requirement for oriental river prawn (*Macrobrachium nipponense*) was assessed, and its impact on agonistic behavior was evaluated in this study. An 8-week feeding trial was implemented with six different tryptophan levels: 0.07%, 0.17%, 0.29%, 0.39%, 0.52%, and 0.64%. The results showed that the highest specific growth rate (SGR) was observed in prawns fed with 0.29% tryptophan. Furthermore, body protein deposition (BPD) and protein efficiency ratio (PER) were significantly elevated in the 0.39% tryptophan group than those in the 0.07% tryptophan group, while the feed conversion rate (FCR) was lowest in the 0.39% tryptophan group. Based on quadratic regression analysis of SGR, BPD, FCR, and PER, the optimal tryptophan requirement was determined to be 0.35%–0.39% of dry matter, 0.90%–1.01% of crude protein. In the 0.52% tryptophan group, crude protein and lipid contents were improved, while ash content was the lowest. The total protein (TP), cholesterol, and urea nitrogen (UN) contents in the hemolymph were observed to be the highest levels in the 0.64% tryptophan group, although there was a reduction in triglyceride (TG) and glucose (GLU) levels. Muscle *tor* gene expression was the highest at 0.39% tryptophan, with *atf4* and *atf3* expressions suppressed. Hepatopancreas *tor* and *cpt1* gene mRNA peaked at 0.29% tryptophan, while *acc*, *fas*, and *atf4* genes were inhibited. Following the feeding trial, prawns fed diets with 0.07%, 0.29%, and 0.64% tryptophan were randomly selected for the detection of agonistic behaviors. Prawns fed 0.29% and 0.64% tryptophan showed less aggression than those fed 0.07%. Serotonin levels were highest in the 0.64% group, followed by 0.29%, and lowest in 0.07%. The *5-ht1b* gene expression was significantly increased in the 0.64% tryptophan group compared to the 0.29% and 0.07% tryptophan groups. Both serotonin levels and *5-ht1b* expression showed the same significant difference before and after fighting behavior, with increases observed postfight across all treatments. In conclusion, the optimal dietary tryptophan requirement for the oriental river prawn was estimated to be between 0.35% and 0.39% of dry matter (0.90% and 1.01% of crude protein), which enhanced growth performance and effectively reduced agonistic behavior.

## 1. Introduction

The requirement for essential amino acids is a critical consideration in the formulation of commercial aquafeeds. Tryptophan, widely recognized as the third limiting essential amino acid, has garnered significant research attention. Research has established tryptophan requirements for key species, including the Chinese mitten crab (*Eriocheir sinensis*) [1] and the tiger shrimp (*Penaeus monodon*) [2]. Optimal levels of tryptophan supplementation improved survival rates in Chinese mitten crab [3] and giant river prawns (*Macrobrachium rosenbergii*) [4], as well as enhanced growth performance in mud crabs (*Scylla serrata*) [5]. Tryptophan further boosted the immunity and antioxidant capacity of the Chinese mitten crab [6] and mitigated low-salinity stress in *Fenneropenaeus chinensis* [7]. Therefore, it is necessary to determine the precise tryptophan requirement of oriental river prawn (*Macrobrachium nipponense*) to develop optimized compound feed.

Molecular biological techniques are now extensively employed to assess metabolic processes beyond conventional biochemical indices and enzyme activities. Genes, such as acetyl-CoA carboxylase (ACC), fatty acid synthase (FAS), and carnitine palmitoyltransferase 1 (CPT1), play pivotal roles in lipid synthesis, fatty acid oxidation, and glucose (GLU)-lipid metabolism [8]. Numerous studies demonstrated that amino acids modulated the expression of lipid-metabolism genes. For instance, in the swimming crab (*Portunus trituberculatus*), dietary isoleucine supplementation significantly downregulated *fas* and *acc* expression, suppressing lipogenesis [9]. Furthermore, activated transcription factor 3 (ATF3) and activated transcription factor 4 (ATF4) are commonly activated under amino acid deprivation or imbalance [10]. Research indicated that the relative expression of the *atf4* gene was elevated under fishmeal-reduced conditions [11], implicating its role in nutrient sensing. The target of rapamycin (TOR) kinase pathway further integrates these signals, potentially activating or inhibiting downstream metabolic genes. However, the specific regulatory mechanisms and the precise interactions underlying these processes remain to be elucidated.

Crustaceans in aquaculture environments often exhibit strong aggression when competing for food, territory, and mates [12]. This aggression frequently results in injury or death, reducing survival rates, lowering yields, and causing significant economic losses. Furthermore, dietary tryptophan has been shown to effectively inhibit agonistic behavior in Chinese mitten crab [12]. As an essential amino acid, tryptophan serves not only as a protein building block but also as a precursor for 5-hydroxytryptamine (5-HT), a neurotransmitter involved in the regulation of behavior from emotional responses in mammals to aggression in crustaceans [13, 14]. Dietary tryptophan supplementation has been found to increase 5-HT levels and suppress agonistic behavior in Chinese mitten crabs [12]. Similarly, in juvenile mud crabs, elevated tryptophan levels reduced aggression and improved survival by modulating serotonergic pathways [5]. Moreover, 5-HT and its receptor genes were involved in agonistic behavior in *M. rosenbergii* [15]. These findings indicated that optimal tryptophan supplementation may help mitigate agonistic behaviors in aquatic species. However, no studies have specifically investigated the potential of dietary tryptophan to reduce agonistic behavior in the oriental river prawn. Addressing these knowledge gaps is crucial for optimizing feed formulations and enhancing aquaculture sustainability.

Oriental river prawn, a principal economic species of freshwater prawn in China, is characterized by rapid growth, a short cultivation cycle, and an omnivorous diet [11]. With the ongoing expansion of oriental river prawn culture and the increased utilization of formulated feeds, substantial research has been conducted on commercial feed formulations. Studies have investigated dietary protein [16, 17], lipid composition [18], and carbohydrate [19] in oriental river prawn. Research on amino acid requirements has predominantly concentrated on threonine [20] and arginine [21], with no reported data on tryptophan. Furthermore, the agonistic behavior of crustaceans presents a significant challenge in aquaculture. As a typical territorial crustacean, the oriental river prawn frequently exhibits agonistic behavior in high-density farming environments, leading to limb injuries and reduced survival rates [22].

Consequently, it was hypothesized that dietary tryptophan would simultaneously enhance growth performance and attenuate aggression in the oriental river prawn. Then, the tryptophan requirement for the oriental river prawn was assessed by growth performance in this study. Physiological parameters, including muscle amino acid profiles and hemolymph biochemical indices, and key genes regulating protein and lipid metabolism, were detected to further illustrate the mechanisms. Furthermore, agonistic behavior, serotonin, and 5-HT1B receptor were quantified to elucidate the aggression modulation in this study. It was expected to provide a theoretical foundation for formulating cost-effective aquafeeds that improve growth and reduce aggression-induced mortality in intensive aquaculture systems, while also establishing novel insights into amino acid-mediated behavioral regulation in crustaceans.

## 2. Materials and Methods

### 2.1. Experimental Diet Preparation

Six isonitrogenous (38% crude protein) and isolipidic (7.7% crude lipid) diets were formulated using fish meal, gelatin, corn gluten meal, and crystalline amino acid mixture as primary protein sources. The amino acid profiles of fishmeal, gelatin, and corn gluten were first determined. A crystalline amino acid mixture was then supplemented to meet all amino acid requirements except tryptophan, with glycine added to maintain nitrogen equilibrium across differential tryptophan levels. The lipid consisted of fish oil and soybean oil (1:1, v/v), while α-starch served as a carbohydrate source. Tryptophan was supplemented at graded levels in each group, which were 0%, 0.15%, 0.30%, 0.45%, 0.60%, and 0.75%, corresponding to actual tryptophan concentrations of 0.07%, 0.17%, 0.29%, 0.39%, 0.52%, and 0.64%, respectively (Table 1). All crystalline amino acids, including tryptophan, were bought from Feer Co., Ltd. (Shanghai, China) with a purity above 99%. All ingredients were ground, passed through an 80-mesh sieve, and precisely weighed according to the formula. The dry ingredients were initially mixed using step-by-step amplification first, and then thoroughly blended with water and oil. The sinking pellets (1.0 mm) were processed with a twin-screw extruder (F-26, Guangzhou Huazhong Optical Mechanical and Electrical Technology Co., Ltd., Guangzhou, China) and air-dried at room temperature until the moisture content was about 10%. The formulated diets were subsequently vacuum-sealed and stored at −15°C for future use.

### 2.2. Experimental Animal and Feeding Trial

The healthy oriental river prawns, characterized by a smooth body surface without external injuries, intact appendages, active behavior, rapid responsiveness to stimuli, and normal feeding, were sourced from the Dapu Breeding Farm of Freshwater Fisheries Research Center, CAFS (Wuxi, China). Prawns were acclimated to the rearing system for 2 weeks with the commercial diet (36% crude protein and 6% crude lipid) first. Prawns (initial average body-weight 0.20 ± 0.01 g) were randomly allocated to 50 individuals in each tank. A total of 24 circular fiberglass tanks (*φ*1.5 m, 800 L water per tank) were randomly assigned to six experiment diets with four replicate tanks per diet. Each tank was a single cylindrical unit with static water, which was equipped with aerators to ensure adequate dissolved oxygen for the prawns. The trial operated under natural light. Artificial plastic leaves were served as shelters for prawns in the tanks. To maintain the water quality, feces and residual diet were cleaned three times daily, and ~1/10 of the water was changed every 3 days. Water temperature was between 27 and 30 °C, pH 7.2–7.8, ammonia nitrogen < 0.02 mg L^−1^, and dissolved oxygen > 6.5 mg L^−1^.

Prawns were hand-fed thrice daily at consistent intervals: 7:30–8:00, 11:30–12:00, and 16:30–17:00. The feeding amount was ~2%–5% of the prawns' biomass in the tanks. Uneaten feed was removed 1 h postfeeding, dried, and weighed for calculation. The weight of the feed administered daily was recorded. The feeding trial lasted for 8 weeks.

### 2.3. Sample Collection

Prior to sample collection, prawns were fasted for 24 h. All prawns in every tank were counted and weighed to assess growth performance. About 12 prawns per tank were randomly chosen for sample collection. They were first put into the mixture of water and ice for anesthesia. Hemolymph was collected from the cardiac celom using an anticoagulant solution (1:1 v/v; anticoagulant composition: 450 mM NaCl, 10 mM KCl, 10 mM EDTA-Na_2_, 10 mM HEPES, pH 7.3). They were held at 4 °C for 2 h before being centrifuged at 3000 g for 10 min at 4 °C. The supernatant was then stored at −20°C until biochemical analysis was performed. Three pooled hemolymph samples were obtained per tank. Following hemolymph collection, the prawns were placed on ice trays for dissection. The hepatopancreas and muscle tissues were removed and promptly preserved in liquid nitrogen for subsequent RT-PCR analysis. Additionally, pooled muscle samples were obtained to assess the muscle amino acid profile. Another eight individuals were randomly chosen from one tank, and then stored at −20 °C as a pooled sample for subsequent whole-body composition analysis.

### 2.4. Proximate Composition and Amino Acid Profile Analysis of Diet and Prawns

The proximate composition of experimental diets and prawns was determined according to the established methods outlined by the AOAC (2005) [23]. Moisture content (930.15) was measured by drying samples at 105 °C to a constant weight. The determination of crude protein (*N* × 6.25; 954.01) was conducted by the Kjeldahl method postacid digestion. The crude lipid content (920.39) was assessed through the ether-extraction method employing a Soxhlet system HT6, while ash content (942.05) was quantified by incineration in a muffle furnace at 560 °C for about 5 h.

Amino acid concentrations in the diets (Table 2) and muscle tissues were analyzed at the Public Testing Laboratory (Jiangnan University School of Food Science and Technology, Wuxi, China) according to the national standard method (GB/T 18246-2019) [21]. For amino acid content analysis, samples were accurately weighed and hydrolyzed at 110 °C in 6 mol L^−1^ HCl for 24 h. After pretreatment, all samples were analyzed using the amino acid reagent organizer (S7130, Sykam Technologies Co., Ltd., Germany). To ascertain tryptophan content in diets and muscle, the samples were hydrolyzed using 4 mol L^−1^ lithium hydroxide according to the national standard method (GB/T 15400). The separation was conducted via high-performance liquid chromatography (Waters Technology Shanghai Co., Ltd., Shanghai, China), with detection facilitated by ultraviolet or fluorescence detectors, and quantified by the external standard method.

### 2.5. Hemolymph Biochemical Indices Determination

The concentrations of total protein (TP), total cholesterol (TC), triglyceride (TG), GLU, and urea nitrogen (UN) in hemolymph were measured by the colorimetric test kits (Mindray Bio Medical Co., Ltd., Shenzhen, China) on an auto-biochemical analyzer (Mindray BS-400, Mindray Bio Medical Co., Ltd., Shenzhen, China) according to the manufacturer's protocol, described as Worlanyo [20]. The assays employed enzymatic colorimetric principles: TP was determined via the biuret method; TC and TG utilized oxidase/peroxidase-coupled reactions (cholesterol oxidase for TC; glycerol-3-phosphate oxidase for TG); GLU was quantified by the GLU oxidase method; and UN was measured via urease-mediated hydrolysis with glutamate dehydrogenase.

### 2.6. RT-PCR

Total RNA was extracted from hepatopancreas and muscle samples using RNAiso plus kit (Takara, Dalian, China) as described by Zhou [11]. The quality and quantity of RNA were assessed at 260 and 280 nm using a NanoDrop spectrophotometer (Thermo Scientific, USA). Additionally, RNA integrity was confirmed through 1% agarose gel electrophoresis. Total RNA samples were diluted to a concentration of 500 ng and then used for cDNA synthesis employing the ExScript RT-PCR kit (Takara Co., Ltd., Japan).

Primers, detailed in Table 3, were synthesized (Generay Biotechnology Co., Ltd., Shanghai, China). The cDNA samples were subjected to analysis by RT-PCR (Bio-Rad, USA) in conjunction with the TB Green Ⅱ Fluorescence Kit (Takara Co., Ltd., Japan). The reaction solution and thermal cycling conditions were prepared in accordance with the manufacturer's protocol as outlined by Zhou et al. [11]. Relative expression levels for genes were calculated by the 2^−ΔΔCT^ method [26] with β-actin as a reference gene. The mean values from three distinct individual samples in the same tank were used in subsequent statistical analysis.

### 2.7. Fighting Behavior Observation and Detection

Following the feeding trial, prawns fed with 0.07%, 0.29% and 0.64% tryptophan were randomly selected for aggression observation. Two prawns from the same tank, matched for size, body weight, and complete limbs, were paired for the observation of the fighting behavior. They were placed in a transparent glass arena (20 cm × 15 cm × 10 cm) filled to a depth of 5 cm with water. After a 5-min acclimation period separated by a partition, the separation was removed to record the behavior of prawns by a digital camera for a 1-h period. A digital camera (PROMOTION YOUR POWER, Shenzhen, China) was employed to record the aggressive behaviors between the two prawns. Based on established crustacean aggression ethograms [27], aggressive behaviors were categorized into approach, touch, and fight. Approach is identified as one prawn move directly toward another using its second pereiopods without physical contact, touch means physical contact with the second pereiopods against any body part of the opponent, while fight is identified as active use of the second pereiopods to pinch or push opponents. The total aggressive behaviors and the cumulative duration of fighting were quantified in 1 h. To eliminate residual chemical cues, the glass arena was thoroughly rinsed and refilled with fresh tap water between different experiments. Three independent observations of aggressive behaviors were conducted for each tank.

Hemolymph, as well as the hepatopancreas, were collected following the same protocol as previously described before and after the aggressive behavior observation trial. The concentration of 5-HT in hemolymph was quantified by 5-HT ELISA Kit (Beijing Jinzhiyan Biotechnology Co., Ltd., Beijing, China) according to the protocol, while the expression of *5-ht1b* in the hepatopancreas was assessed via RT-PCR. The primer for *5-ht1b* was designed using Primer-Blast at NCBI based on the partial cDNA sequences obtained from the *M. nipponense* transcriptome analysis [28, 29].

### 2.8. Statistical Analysis

The mean value for each parameter per tank was calculated first, and then submitted to SPSS 19.0 for statistical analysis. If the variance was homogeneous, one-way analysis of variance (ANOVA) was performed. Significant differences between treatments (*p* < 0.05) were identified using Duncan's multiple range test. Additionally, a second-degree polynomial regression analysis was conducted in Origin 2024b to determine the optimal tryptophan level. Gene expression figures were generated using GraphPad Prism 8.0. All results are presented as mean ± standard error (mean ± SE).

## 3. Results

### 3.1. Growth Performance and Feed Utilization

Dietary tryptophan had a significant effect on the growth and feed utilization of oriental river prawns (Table 4). At 0.29% tryptophan, both the survival rate and the average final body weight (FBW) of prawns reached peaks without a significant difference (*p* > 0.05). The specific growth rate (SGR) and weight gain rate (WGR) of prawns fed with tryptophan supplementation were higher than those in the control group (*p* < 0.05). Notably, prawns fed with 0.29% tryptophan exhibited the highest SGR and WGR, which were significantly higher than those observed in 0.64% tryptophan treatment (*p* < 0.05). Additionally, the feed conversion rate (FCR) was the lowest in prawns fed with 0.39% tryptophan, compared to those fed with 0.17% and 0.29% tryptophan, without a significant difference (*p* > 0.05), and was significantly lower than those fed with the basal diet and 0.64% tryptophan (*p* < 0.05). Contrarily, the protein efficiency ratio (PER) increased with the elevation of dietary tryptophan levels from 0.07% to 0.39%, with the maximum observed in the 0.39% tryptophan treatment, which was significantly higher than the 0.07% and 0.64% tryptophan groups. Notably, except for the prawns fed diets containing 0.29% tryptophan, the body protein deposition (BPD) in prawns fed 0.39% tryptophan was significantly higher than that in other treatments.

**Figure 1:**
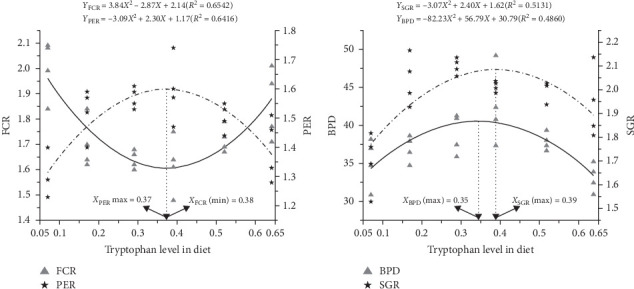
Optimal tryptophan in the diet of oriental river prawn based on polynomial regression analysis of FCR, PER, SGR, and BPD. BPD, body protein deposition; FCR, feed conversion rate; PER, protein efficiency ratio; SGR, specific growth ratio.

Linear, quadratic, and cubic models were employed to assess the trends in growth and feed utilization with increasing tryptophan levels. There were extremely significant quadratic and cubic trends for SGR, WGR, FCR, PER, and BPD (*p* < 0.01). According to the second-degree polynomial regression analysis, the optimal dietary tryptophan requirements were identified as 0.38%, 0.37%, 0.39%, and 0.35% of dry matter for FCR, PER, SGR, and BPD, respectively (Figure 1), which corresponded to 0.98%, 0.95%, 1.01%, and 0.90% of crude protein. The coefficients of these quadratic regression models were determined as follows: *R*^2^ = 0.65 for FCR, 0.64 for PER, 0.51 for SGR, and 0.49 for BPD.

### 3.2. Whole-Body Composition and Muscle Amino Acid Profile

Dietary tryptophan did not significantly affect the moisture content of oriental river prawns (*p* > 0.05) (Table 5). But the crude protein, crude lipid, and ash contents of prawns in 0.39% tryptophan treatment were the lowest among all treatments. Specifically, the crude protein content in the 0.39% tryptophan treatment was lower than that in other treatments (*p* < 0.05). The crude lipid and ash contents in prawns fed with 0.64% tryptophan were higher than those in the 0.39% tryptophan treatment (*p* < 0.05).

Apart from tryptophan, dietary tryptophan did not have significant effects on muscle amino acid profile (*p* > 0.05) (Table 6). As the levels of dietary tryptophan increased, the concentration of tryptophan in muscle tissue also increased, reaching its peak in the group fed with 0.64% tryptophan (*p* < 0.05). The ratio of essential amino acids and nonessential amino acids of prawns fed with 0.39% tryptophan was the lowest among all treatments and was lower than that of prawns fed with the basal diet and 0.64% tryptophan (*p* < 0.05).

### 3.3. Hemolymph Biochemical Parameters

Dietary tryptophan exhibited a significant impact on the hemolymph biochemical index (*p* < 0.05) (Table 7). The TP and UN contents were maximized in the 0.64% tryptophan treatment, with the TP content significantly exceeding that observed in the 0.07% tryptophan treatment, and the UN content significantly surpassing those in the 0.29% and 0.39% tryptophan treatments (*p* < 0.05). The TG level in the 0.29% tryptophan treatment was higher compared to the 0.17%, 0.52%, and 0.64% tryptophan treatments (*p* < 0.05), although no significant difference was observed among the remaining treatments (*p* > 0.05). Furthermore, the TC content in 0.64% tryptophan treatment was higher than that in 0.17% tryptophan treatment (*p* < 0.05). The GLU content in the hemolymph was found to be the highest in the basal diet and lowest in the 0.64% tryptophan treatment, with the GLU content in the control group being significantly higher than that in all other treatments, except for the 0.29% tryptophan treatment (*p* < 0.05).

### 3.4. Relative Gene Expression in Hepatopancreas and Muscle

**Figure 2:**
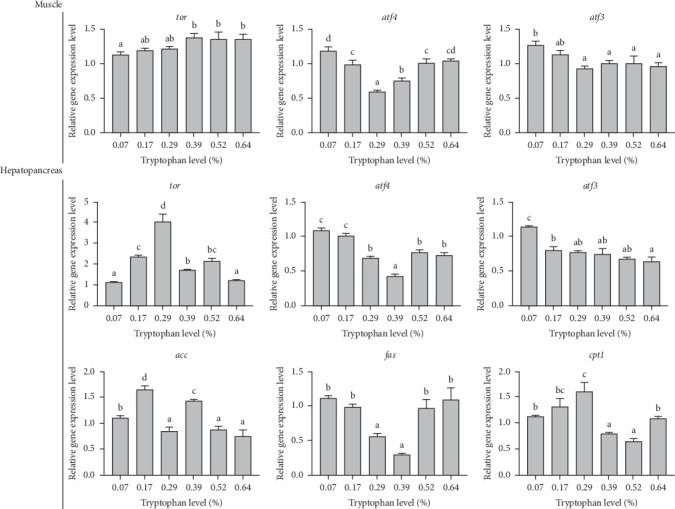
Gene expression in muscle and hepatopancreas of oriental river prawn. Different superscript letter means significantly different, determined by the Duncan test (*p* < 0.05).

Dietary tryptophan significantly affected the expression of genes associated with protein metabolism in hepatopancreas and muscle tissues of the oriental river prawn (*p* < 0.05) (Figure 2). In muscle tissue, the relative expression levels of the *tor* gene in the control group were significantly lower than those in the 0.39%, 0.52%, and 0.64% tryptophan groups (*p* < 0.05), with the highest mRNA level observed in the 0.39% tryptophan group. Conversely, the mRNA levels of the *atf4* and *atf3* genes were the highest in the control group. Specifically, the *atf4* mRNA level in the muscle tissue was significantly reduced in the 0.29% tryptophan group compared to other groups (*p* < 0.05). Additionally, the relative expression of the *atf3* gene in prawns fed with 0.07% tryptophan was significantly higher than that in all other groups, except for the 0.17% tryptophan group (*p* < 0.05).

In hepatopancreas, the highest relative expression of the *tor* gene was found in prawns fed with 0.29% dietary tryptophan, which was significantly higher compared to other treatments (*p* < 0.05). Conversely, the lowest mRNA level of the *atf4* gene was recorded in the 0.39% tryptophan treatment. The mRNA level of the *atf3* gene in the basal diet was significantly higher than that in other treatments (*p* < 0.05). The relative expression of the *acc* gene reached its peak in the 0.17% tryptophan group, displaying a significantly higher level than that in other tryptophan treatments (*p* < 0.05). Notably, the mRNA level of the *fas* gene was markedly reduced in the 0.29% and 0.39% tryptophan groups. In contrast, the relative expression of the *cpt1* gene exhibited the maximum in the 0.29% tryptophan group, significantly surpassing the levels observed in other treatments (*p* < 0.05).

### 3.5. Fighting Behavior Observation and Detection

**Figure 3:**
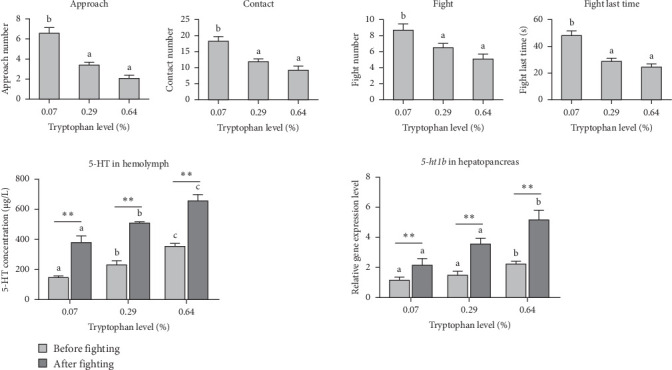
Fighting behavior, 5-HT content in hemolymph, and relative expression of *5-ht1b* in hepatopancreas before and after fighting of oriental river prawn. Different superscript letter means significantly different, determined by the Duncan test (*p* < 0.05). *⁣*^*∗∗*^means significant difference between before and after fighting determined by *t*-test (*p* < 0.01).

Dietary tryptophan exerted a significant effect on the agonistic behavior of oriental river prawn (*p* < 0.05) (Figure 3). An increase in tryptophan resulted in a notable reduction in approach, contact, and fighting behaviors, as well as the duration of fighting among prawns (*p* < 0.05). Specifically, prawns fed with 0.29% and 0.64% tryptophan exhibited significantly lower levels of fighting behavior compared to the basal diet (*p* < 0.05). Additionally, the concentration of 5-HT in the hemolymph of prawns increased significantly with increasing dietary tryptophan both prior to and following fighting behavior (*p* < 0.05). The 5-HT levels in the hemolymph postfighting were significantly higher compared to prefighting levels across all treatments (*p* < 0.05). Furthermore, the relative expression levels of the *5-ht1b* gene in the hepatopancreas of prawns fed 0.64% tryptophan were significantly higher than those in prawns fed 0.07% and 0.29% tryptophan, both prior to and following fighting behavior (*p* < 0.05). The postfighting relative expression level of the *5-ht1b* gene in the hepatopancreas was markedly higher than the prefighting level (*p* < 0.05).

## 4. Discussion

### 4.1. Growth Performance and Nutrient Utilization

Tryptophan, an essential amino acid that crustaceans are unable to synthesize, plays a crucial role in promoting growth and maintaining physiological equilibrium in aquatic animals [30]. In this study, the oriental river prawns fed a diet with optimal tryptophan, 0.35%–0.39% of dry matter (0.90%–1.01% of crude protein), improved the growth. This finding aligned with reports of improved growth performance in various crustaceans, including Chinese mitten crab [1], tiger shrimp [2], and Pacific white shrimp (*Litopenaeus vannamei*) [31]. Quadratic regression analysis revealed that tryptophan levels exceeding 0.39% did not further enhance growth, suggesting a threshold effect. This finding was consistent with observations in other decapod species, such as mud crab (*Scylla paramamosain*) [32] and Pacific white shrimp [33], where supplemental tryptophan beyond optimal levels also failed to enhance growth. This plateau is likely due to metabolic trade-offs between protein synthesis and neurotransmitter production [34]. Moreover, feed utilization was maximized at the optimal tryptophan requirement in this study, which was consistent with observations in Pacific white shrimp [31] and *Marsupenaeus japonicus* [35]. Excessive dietary tryptophan reduced protein efficiency in this study. This effect might be because of tryptophan's antagonism with other amino acids, analogous to the antagonistic interaction between dietary lysine and arginine in black sea bream (*Acanthopagrus schlegelii*) [36]. However, different tryptophan requirements were observed in fish species such as red drum (*Sciaenops ocellatus*) at 0.28% [37] and stinging catfish (*Heteropneustes fossilis*) at 0.24%–0.27% [38]. This variation might be attributed to the unique physiological demands of decapod crustaceans, which require multiple molting events throughout their growth cycle. These multiple molting events increased their amino acid requirements to support exoskeleton regeneration and tissue synthesis [30].

In this study, dietary tryptophan had a significant effect on the body composition of the oriental river prawn. This consisted with findings in Pacific white shrimp [31]. The observed decrease in crude protein content of whole-body composition at high dietary tryptophan group in this study suggested a potential reduction in overall protein utilization efficiency or an enhancement of protein catabolism. When the dietary amino acid ratio became imbalanced, particularly with a significant excess of a single amino acid such as tryptophan, the organism might be unable to effectively utilize other amino acids for protein synthesis. The unbalanced amino acid profile in this study might be partly supported by the *atf4* upregulation in hepatopancreas. The unused amino acids were consequently catabolized for energy production or converted into fat stores. This was also partly supported by the *fas* upregulation. This metabolic process consumed dietary protein, leading to a reduction in the net crude protein deposited in body tissues. Additionally, excess tryptophan might alter the balance between protein synthesis and degradation, changing protein turnover, by influencing regulatory pathways. This interpretation was further supported by the observed *tor* downregulation in the hepatopancreas.

Notably, muscle tryptophan content increased proportionally with dietary tryptophan, while other amino acids remained stable in this study. It contrasted with reports in other crustaceans. Dietary tryptophan influenced proline levels in Chinese mitten crab [12] and significantly altered threonine, methionine, and leucine besides tryptophan in *L. vannamei* [31]. This discrepancy might reflect species-specific variations in nutrient utilization priorities across developmental stages. Alternatively, the different tryptophan effects on muscle amino acids might originate from intestinal absorption saturation and feedback regulation mechanisms that maintain systemic amino acid homeostasis. Further investigation was needed to illustrate the underlying physiological mechanism.

Dietary tryptophan levels significantly influenced hemolymph biochemical parameters in this study. The prawns fed excessive dietary tryptophan exhibited the high UN level, which was consistent with reports of threonine requirement in *M. nipponense* [20] and tryptophan requirement in *L. vannamei* [31]. This response might likely be caused by enhanced protein turnover. Excessive tryptophan is likely to be preferentially utilized in protein synthesis, while also accelerating protein catabolism. Then the amino acid degradation promoted nitrogenous wastes such as urea, leading to elevated UN concentrations [39]. Moreover, hemolymph TG and GLU concentrations decreased in prawns fed with excessive tryptophan, which was in contrast to reports from threonine requirement in *M. nipponense* [20] and tryptophan requirement in *L. vannamei* [31]. This might reflect the altered energy metabolism, where surplus amino acids partially substitute for energy substrates. Further study was needed to confirm this hypothesis.

While this 8-week trial elucidated the acute metabolic responses to high tryptophan supplementation, it cannot capture potential long-term physiological adaptations or chronic effects. Long-term high tryptophan might increase amino acid metabolism, elevate UN, and cause oxidative damage in the hepatopancreas. Moreover, high tryptophan persistently elevated serotonin, which might cause negative effects on hormone secretion, which might affect the molting and ovarian maturation. These hypotheses required validation through longitudinal studies over full life cycles.

### 4.2. Molecular Regulation of Metabolism

Existing research has demonstrated that the TOR gene in aquatic animals primarily regulates growth and metabolism by promoting protein synthesis and influencing growth rates [40]. The *tor* gene was activated in the muscle and hepatopancreas tissues by optimal tryptophan in 0.29% and 0.39% tryptophan treatments, which might partly explain the observed enhanced growth performance. Similar findings had been reported in crustaceans such as the Chinese mitten crab [6] and swimming crabs [41]. Further studies have shown that TOR activation in crustaceans enhances protein synthesis by phosphorylating downstream genes like ribosomal protein S6 kinase (S6K) [41, 42]. It was found that the *atf4* and *atf3* genes were inhibited in the 0.29% tryptophan group in this study. Previous studies had indicated that ATF4 primarily responds to amino acid deprivation [10], while ATF3 modulates stress-induced metabolic adaptations [43]. ATF4 and ATF3 would be activated under conditions of amino acid restriction [10], whereas their cosuppression in optimal groups indicates balanced amino acid status, which was proved in this study.

In the hepatopancreas, suitable tryptophan downregulated *fas* and *acc* expression, indicating suppression of de novo lipogenesis, which was aligned with the findings in Pacific white shrimp [44]. As rate-limiting enzymes in fatty acid biosynthesis, *acc* encodes ACC, catalyzing the conversion of acetyl-CoA to malonyl-CoA (the initial step), and *fas* encodes FAS, elongating malonyl-CoA into long-chain fatty acids [45]. Their coordinated downregulation reflects a metabolic shift from lipid storage to energy mobilization [41]. Concurrently, *cpt1* mRNA levels increased, enhancing mitochondrial import of fatty acids for β-oxidation. Carnitine palmitoyl-transferase 1 is a key enzyme for this process. The inverse regulation of *fas/acc* and *cpt1* genes may suggest the breakdown of stored lipids to meet energy demands, redirecting energy from lipid accumulation toward protein synthesis and somatic growth, thereby supporting improved growth performance [46].

### 4.3. Agonistic Behavior and Serotonergic Signaling

Tryptophan may inhibit agonistic behavior by increasing 5-HT concentrations in the brain, as confirmed in *L. vannamei* [33]. In the present study, dietary tryptophan supplementation effectively suppressed fighting behavior and reduced aggression duration in oriental river prawns. These findings were consistent with observations in meager (*Argyrosomus regius*) [47] and mud crabs [5]. Tryptophan may inhibit agonistic behavior by increasing 5-HT concentrations in the brain, as confirmed in *L. vannamei* [33]. Furthermore, dietary tryptophan supplementation has been demonstrated to significantly elevate 5-HT levels and upregulate the expression of its receptor *5-ht1b*, with the 0.64% tryptophan group exhibiting the peak values. It was consistent with previous studies on crayfish (*Astacus leptodactylus* Eschscholtz) [48] and mud crab [5], where elevated dietary tryptophan was associated with increased 5-HT content and suppressed the agonistic behaviors. Further investigations have explored the role of 5-HT and its receptor 5-HT1A in modulating agonistic behavior, revealing that activation of 5-HT1A could inhibit agonistic behavior in Siamese fighting fish (*Betta splendens*) [49].

Furthermore, a nonlinear behavioral response to tryptophan supplementation was observed in this study. It might originate from negative feedback regulation mediated by 5-HT1B receptors on serotonin (5-HT) neurotransmission. The 5-HT biosynthesis pathway involves tryptophan transport via the serotonin transporter (SERT) followed by rate-limiting hydroxylation catalyzed by tryptophan hydroxylase (TPH) [50, 51]. Critically, activation of presynaptic 5-HT1B suppresses TPH activity through a paradoxical feedback mechanism, ultimately attenuating tryptophan conversion to 5-HT. However, a converse result has been reported that increased 5-HT levels may stimulate aggression in swimming crabs (*Portunus trituberculatus*) [52] and crayfish (*Procambarus clarkii*) [53]. Likely due to species-specific receptor subtypes: 5-HT1B mediates inhibition in prawns, while 5-HT2 receptors may drive aggression in Chinese mitten crab [54], with *5-ht2* upregulation enhancing signal transduction postfight. The specific behavioral functions mediated by different types of 5-HT receptors might vary across species, and thus, further in-depth studies are warranted to elucidate these differences.

Since 0.35%−0.39% tryptophan was identified as optimal for growth and feed utilization in oriental river prawn in this study, it was essential to assess the feasibility of incorporating this level into commercial feed formulations. While tryptophan is naturally present in common feed ingredients such as fishmeal, soybean meal, and corn gluten meal, precise consideration of its concentration during formulation is critical. Commercially available L-tryptophan supplements offer a technically viable solution for achieving target levels within standard feed manufacturing processes. This approach ensures nutritional balance by adhering to ideal protein profiles and maintains compliance with regulatory standards without compromising feed physical quality.

## 5. Conclusion

In summary, this study identified that the optimal dietary tryptophan requirement for the oriental river prawns is 0.35%−0.39% of dry matter (0.9%−1.0% of crude protein) through quadratic regression analysis based on SGR, BPD, FCR, and PER. Furthermore, supplementation with 0.29% and 0.64% tryptophan significantly mitigated agonistic behaviors, including approach, contact, fighting, and fighting duration, while concurrently increasing hemolymph 5-HT content and hepatopancreatic *5-ht1b* gene expression, with notable effects observed in the 0.64% tryptophan supplementation level. These findings suggested that dietary tryptophan not only promoted growth performance but also reduced agonistic behavior. Consequently, precise optimization of tryptophan levels during feed formulation is essential for a cost-effective aquafeed. Collectively, this research established a scientific framework for formulating aquafeeds that simultaneously improved growth performance and mitigated aggression in intensive prawn culture systems.

## Figures and Tables

**Table 1:** Feed formulation and proximate composition of the experimental feeds.

Ingredients	Dietary tryptophan levels (%)					
	0.07	0.17	0.29	0.39	0.52	0.64
Fish meal	15.00	15.00	15.00	15.00	15.00	15.00
Gelatin	5.00	5.00	5.00	5.00	5.00	5.00
Corn gluten meal	10.00	10.00	10.00	10.00	10.00	10.00
Amino acid mix^a^	18.44	18.44	18.44	18.44	18.44	18.44
Oil^b^	4.00	4.00	4.00	4.00	4.00	4.00
α-starch	33.61	33.61	33.61	33.61	33.61	33.61
Carboxymethyl cellulose	3.00	3.00	3.00	3.00	3.00	3.00
Premix^c^	2.00	2.00	2.00	2.00	2.00	2.00
Others^d^	8.20	8.20	8.20	8.20	8.20	8.20
L-glycine	0.75	0.60	0.45	0.30	0.15	0.00
L-tryptophan^e^	0.00	0.15	0.30	0.45	0.60	0.75
Proximate composition of experimental diet (percentage of dry matter)						
Crude protein	38.88	38.91	38.89	38.81	38.83	38.80
Crude lipid	7.67	7.66	7.57	7.79	7.69	7.60
Ash	8.03	7.84	8.01	7.79	7.93	8.11
Tryptophan (percentage of dry diet)	0.07	0.17	0.29	0.39	0.52	0.64
Tryptophan (percentage of crude protein)	0.17	0.44	0.74	1.01	1.34	1.66

^a^Amino acid mix (18.44 g) included essential amino acids, L-arginine, 1.77 g; L-histidine, 0.44 g; L-isoleucine, 1.05 g; L-leucine, 0.97 g; L-lysine, 1.98 g; DL-methionine, 0.58 g; L-phenylalanine, 0.70 g; L-threonine, 0.58 g; L-valine, 0.89 g; and nonessential amino acids, L-aspartic acid, 2.79 g; L-serine, 0.30 g; L-glycine, 1.66 g; L-alanine, 1.29 g; L-cystine, 0.17 g; L-tyrsine, 0.61 g; L-gulmatic acid, 2.66 g; which were all bought from Feer Co., Ltd. (Shanghai, China).

^b^Oil was the mix of fish oil and soybean oil with a volume of 1:1.

^c^Premix (IU, g or mg kg^−1^ of diet): vitamin A, 25,000 IU; vitamin D3, 20,000 IU; vitamin E, 200 mg; vitamin K3, 20 mg; thiamin, 40 mg; riboflavin, 50 mg; calcium pantothenate, 100 mg; pyridoxine HCl, 40 mg; cyanocobalamin, 0.2 mg; biotin, 6mg; folic acid, 20 mg; niacin, 200 mg; inositol, 1000 mg; vitamin C, 2000 mg; choline, 2000 mg; calcium biphosphate, 20 g; sodium chloride, 2.6 g; potassium chloride, 5 g; magnesium sulfate, 2 g; ferrous sulfate, 0.9 g; zinc sulfate, 0.06 g; cupric sulfate, 0.02 g; manganese sulfate, 0.03 g; sodium selenate, 0.02 g; cobalt chloride, 0.05 g; potassium iodide, 0.004 g.

^d^Others included monocalcium phosphate, 3%; soy lecithin, 2.5%; choline chloride, 1%; ascorbic phosphate ester 0.5%; cholesterol, 0.5%; bentonite, 0.7%.

^e^L-tryptophan was bought from Feer Co., Ltd. (Shanghai, China) with the purity above 99%.

**Table 2:** Amino acid composition of experimental diets (g/100 g of dry matter).

Amino acids	Dietary tryptophan levels (%)					
	0.07	0.17	0.29	0.39	0.52	0.64
Essential amino acids						
Arginine	3.21	3.18	3.18	3.21	3.18	3.22
Histidine	1.04	1.04	1.05	1.07	1.05	1.07
Isoleucine	1.97	1.92	1.97	1.96	1.95	1.94
Leucine	3.02	3.02	3.04	3.04	3.00	3.07
Lysine	3.19	3.18	3.19	3.21	3.13	3.17
Methionine	1.12	1.11	1.13	1.11	1.14	1.15
Phenylalanine	1.80	1.82	1.84	1.87	1.78	1.89
Threonine	1.49	1.50	1.47	1.49	1.49	1.52
Valine	2.02	2.04	2.06	2.02	2.04	2.06
Tryptophan	0.07	0.17	0.29	0.39	0.52	0.64
Nonessential amino acids						
Aspartic acid	4.16	4.18	4.09	4.26	4.20	4.21
Serine	1.34	1.35	1.33	1.35	1.33	1.39
Glycine	2.73	2.64	2.47	2.43	2.29	2.19
Alanine	3.02	3.04	3.03	3.03	3.00	3.04
Cystine	0.10	0.10	0.10	0.11	0.08	0.10
Tyrosine	1.18	1.15	1.18	1.24	1.14	1.23
Glutamic acid	5.51	5.51	5.47	5.37	5.48	5.52
Proline	1.92	2.14	1.99	2.11	1.97	1.76
Total	38.04	38.26	38.05	38.40	37.92	38.33

**Table 3:** Primers used for the RT-PCR.

Target genes	Sequence (5′–3′)	Efficiency (%)	References
*tor*	F: AACAAGTCTCGTCCGTGTCC	96.27	Zhou et al. [11]
	R: TTGAGCAGCTTCACGGCTTA		
*atf4*	F: GGCGGTGCAGTTAAACACTC	119.77	Zhou et al. [11]
	R: GTCAGTTTCACCCATGTCGC		
*atf3*	F: GTGAATAGGAGTGCGGGAGG	93.42	Zhou et al. [11]
	R: CTGTACCACCCCGAAGACAC		
*fas*	F: CGGTCAGACAAACTACGGCT	116.52	Zhou et al. [11]
	R: CACTGAATAGCCACCCCAGG		
*g6pdh*	F: CGTGGACCTTTCTTCATTAG	96.05	Ding et al. [24]
	R: ACCATCAACCATTTGAGAAG		
*cpt1*	F: AATTTTTGACTGGCTTCTCC	107.72	Luo et al. [25]
	R: TCCATTCTGGAAATCATCTG		
*acc*	F: CAAGGTCCACTACATGGTCT	112.09	Luo et al. [25]
	R: ACTCTTCCCAAACTCTCTCC		
*5-ht1b*	F: AGTCGGGAATGTGTTCGTCA	100.61	—
	R: AGCTGCAACCAGAAGGTCAG		
*β-actin*	F: GTGCCCATCTACGAGGGTTA	100.44	Luo et al. [25]
	R: CGTCAGGGAGCTCGTAAGAC		

Abbreviations: *5-ht1b*, 5-hydroxytryptamine receptor 1B; *acc*, acetyl-CoA carboxylase; *atf3*, activating transcription factor 3; *atf4*, activating transcription factor 4; *cpt1*, carnitine palmitoyltransferase 1; *fas*, fatty acid synthase; *g6pdh*, glucose 6 phosphate dehydrogenase; *tor*, target of rapamycin.

**Table 4:** Effect of tryptophan on growth performance and feed utilization of oriental river prawn.

Dietary tryptophan levels (%)	SR (%)^1^	FBW (g)^2^	SGR (%)^3^	WGR (%)^4^	FCR^5^	PER^6^	BPD (%)^7^
0.07	74.00 ± 6.38	0.86 ± 0.09	1.70 ± 0.06^a^	229.54 ± 14.28^a^	2.00 ± 0.06^c^	1.29 ± 0.04^a^	35.23 ± 1.61^ab^
0.17	72.50 ± 5.44	0.90 ± 0.12	2.04 ± 0.05^bc^	318.85 ± 15.32^bc^	1.70 ± 0.05^a^	1.52 ± 0.04^bc^	37.00 ± 0.87^ab^
0.29	87.00 ± 3.70	0.96 ± 0.02	2.10 ± 0.04^c^	336.13 ± 5.60^c^	1.64 ± 0.02^a^	1.57 ± 0.02^c^	38.95 ± 1.32^bc^
0.39	84.50 ± 3.86	0.91 ± 0.02	2.02 ± 0.01^bc^	310.34 ± 3.34^bc^	1.62 ± 0.06^a^	1.60 ± 0.06^c^	42.53 ± 2.50^c^
0.52	76.50 ± 4.92	0.94 ± 0.08	1.98 ± 0.02^bc^	301.42 ± 7.32^bc^	1.72 ± 0.03^ab^	1.50 ± 0.02^bc^	37.92 ± 0.58^b^
0.64	77.00 ± 3.32	0.90 ± 0.06	1.94 ± 0.07^b^	290.62 ± 20.34^b^	1.86 ± 0.07^bc^	1.39 ± 0.05^ab^	33.15 ± 0.93^a^
Polynomial contrasts							
*L*	ns	ns	ns	ns	ns	ns	ns
*Q*	ns	ns	*⁣* ^*∗∗∗*^	*⁣* ^*∗∗∗*^	*⁣* ^*∗∗∗*^	*⁣* ^*∗∗∗*^	*⁣* ^*∗∗*^
*C*	ns	ns	*⁣* ^*∗∗∗*^	*⁣* ^*∗∗∗*^	*⁣* ^*∗∗∗*^	*⁣* ^*∗∗∗*^	*⁣* ^*∗∗*^

*Note:* BWi and BWf, mean initial and final body weight (g); BCPi and BCFf, mean initial and final percentage of muscle protein; TF, total amount of diet consumed; CP, percentage of crude protein of the diet. Values are means ± SEM of four replicates. Means in the same column sharing different superscript letters are significantly different, determined by the Duncan test (*p* < 0.05).

Abbreviations: *C*, cubic; *L*, linear; ns, not significant; *Q*, quadratic.

^1^SR, survival = 100 × (final number of prawn/initial number of prawn).

^2^FBW, final body weight (g/prawn) = final tank biomass/final number of prawns.

^3^SGR, specific growth rate (%/d) = 100 × (Ln final wet weight (g) − Ln initial wet weight g)/duration (days).

^4^WGR, weight gain rate (%) = total wet weight gain/initial prawn total weight.

^5^FCR, feed conversion ratio = dry diet fed/wet weight gain.

^6^PER, protein efficiency ratio = wet weight gain/dry protein intake.

^7^BPD, body protein deposition (%) = 100 × (BWf × BCPf) − (BWi × BCPi)/(TF × CP).

*⁣* ^*∗*^ *p* < 0.05.

*⁣* ^*∗∗*^ *p* < 0.01.

*⁣* ^*∗∗∗*^ *p* < 0.001.

**Table 5:** Whole body composition of oriental river prawn (percentage of w.w.).

Analysis proximate	Dietary tryptophan levels (%)					
	0.07	0.17	0.29	0.39	0.52	0.64
Moisture	78.20 ± 0.76	78.55 ± 0.47	77.21 ± 0.55	79.39 ± 0.84	77.31 ± 0.80	78.29 ± 0.27
Crude protein	13.15 ± 0.14^b^	13.33 ± 0.21^b^	14.32 ± 0.13^c^	12.45 ± 0.01^a^	14.30 ± 0.04^c^	13.43 ± 0.24^b^
Crude lipid	3.60 ± 0.35^ab^	3.33 ± 0.03^a^	3.83 ± 0.01^ab^	3.33 ± 0.28^a^	3.92 ± 0.05^b^	4.10 ± 0.26^b^
Ash	5.26 ± 0.23^ab^	5.15 ± 0.95^ab^	5.50 ± 0.19^ab^	4.55 ± 0.18^a^	5.62 ± 0.07^ab^	6.31 ± 0.01^b^

*Note:* Values are means ± SEM of four replicates. Means in the same line with different superscripts are significantly different by the Duncan test (*p* < 0.05). w.w., means wet weight.

**Table 6:** Amino acid profile in muscle of oriental river prawn (g/100 g of fresh prawn muscle).

Amino acids	Dietary tryptophan levels (%)					
	0.07	0.17	0.29	0.39	0.52	0.64
EAA						
Arginine	1.39 ± 0.02	1.52 ± 0.09	1.33 ± 0.15	1.36 ± 0.01	1.39 ± 0.04	1.44 ± 0.08
Histidine	0.39 ± 0.01	0.36 ± 0.01	0.36 ± 0.03	0.36 ± 0.01	0.34 ± 0.01	0.40 ± 0.02
Isoleucine	0.79 ± 0.01	0.80 ± 0.02	0.80 ± 0.07	0.77 ± 0.02	0.78 ± 0.02	0.82 ± 0.02
Leucine	1.37 ± 0.02	1.38 ± 0.02	1.39 ± 0.12	1.34 ± 0.03	1.34 ± 0.03	1.43 ± 0.04
Lysine	1.55 ± 0.01	1.56 ± 0.02	1.56 ± 0.15	1.49 ± 0.05	1.49 ± 0.04	1.62 ± 0.03
Methionine	0.49 ± 0.01	0.49 ± 0.01	0.50 ± 0.05	0.47 ± 0.02	0.47 ± 0.01	0.50 ± 0.01
Phenylalanine	0.75 ± 0.01	0.76 ± 0.02	0.76 ± 0.06	0.72 ± 0.01	0.74 ± 0.01	0.80 ± 0.02
Threonine	0.66 ± 0.01	0.70 ± 0.02	0.66 ± 0.07	0.67 ± 0.01	0.67 ± 0.01	0.71 ± 0.02
Valine	0.79 ± 0.01	0.80 ± 0.02	0.80 ± 0.07	0.77 ± 0.01	0.78 ± 0.02	0.83 ± 0.02
Tryptophan	0.16 ± 0.01^a^	0.15 ± 0.01^a^	0.17 ± 0.01^a^	0.18 ± 0.03^ab^	0.18 ± 0.01^a^	0.21 ± 0.01^b^
ΣEAA	8.32 ± 0.03	8.50 ± 0.23	8.32 ± 0.75	8.10 ± 0.19	8.17 ± 0.18	8.75 ± 0.25
NEAA						
Aspartic acid	1.80 ± 0.02	1.85 ± 0.05	1.84 ± 0.16	1.79 ± 0.03	1.77 ± 0.01	1.93 ± 0.07
Serine	0.65 ± 0.02	0.70 ± 0.03	0.66 ± 0.07	0.67 ± 0.01	0.67 ± 0.01	0.70 ± 0.03
Glycine	1.18 ± 0.02	1.36 ± 0.07	1.20 ± 0.10	1.28 ± 0.04	1.23 ± 0.02	1.26 ± 0.05
Alanine	1.21 ± 0.03	1.21 ± 0.02	1.23 ± 0.10	1.22 ± 0.03	1.23 ± 0.04	1.28 ± 0.02
Cystine	0.20 ± 0.01	0.20 ± 0.01	0.20 ± 0.02	0.20 ± 0.01	0.19 ± 0.00	0.21 ± 0.01
Tyrosine	0.65 ± 0.01	0.66 ± 0.02	0.66 ± 0.06	0.63 ± 0.01	0.64 ± 0.02	0.70 ± 0.02
Glutamic acid	2.76 ± 0.02	2.80 ± 0.05	2.79 ± 0.23	2.70 ± 0.05	2.69 ± 0.05	2.85 ± 0.10
Proline	0.67 ± 0.02	0.64 ± 0.03	0.65 ± 0.05	0.71 ± 0.03	0.68 ± 0.01	0.70 ± 0.03
ΣNEAA	9.12 ± 0.06	9.42 ± 0.24	9.22 ± 0.78	9.20 ± 0.04	9.08 ± 0.15	9.62 ± 0.31
ΣEAA/ΣNEAA	0.91 ± 0.01^b^	0.90 ± 0.01^ab^	0.90 ± 0.01^ab^	0.88 ± 0.02^a^	0.90 ± 0.01^ab^	0.91 ± 0.01^b^

*Note:* Values are means ± SEM of four replicates. Means in the same line with different superscripts are significantly different by the Duncan test (*p* < 0.05).

**Table 7:** Hemolymph biochemical index of the oriental river prawn.

Indices	Dietary tryptophan levels (%)					
	0.07	0.17	0.29	0.39	0.52	0.64
TP (g·L^−1^)	39.69 ± 4.67^a^	40.97 ± 1.00^ab^	45.18 ± 1.69^ab^	41.75 ± 4.79^ab^	42.43 ± 2.86^ab^	48.45 ± 5.56^b^
TC (mmol·L^−1^)	0.46 ± 0.10^ab^	0.36 ± 0.06^a^	0.52 ± 0.05^ab^	0.47 ± 0.03^ab^	0.40 ± 0.09^ab^	0.54 ± 0.14^b^
TG (mmol·L^−1^)	2.24 ± 0.20^ab^	2.03 ± 0.20^a^	2.53 ± 0.30^b^	2.19 ± 0.16^ab^	2.09 ± 0.14^a^	2.07 ± 0.05^a^
GLU (mmol·L^−1^)	4.95 ± 0.55^c^	3.73 ± 0.46^b^	4.21 ± 0.51^bc^	4.10 ± 0.54^b^	3.85 ± 0.30^b^	2.83 ± 0.23^a^
UN (g·L^−1^)	1.21 ± 0.21^ab^	1.11 ± 0.21^ab^	0.96 ± 0.25^a^	0.96 ± 0.13^a^	1.28 ± 0.29^ab^	1.44 ± 0.21^b^

*Note:* Values are means ± SEM of four replicates. Means in the same line with different superscripts are significantly different by the Duncan test (*p* < 0.05). GLU, glucose content; TC, cholesterol content; TG, triglyceride content; TP, total protein content; UN, urea nitrogen content.

## Data Availability

The data that support the findings of this study are available from the corresponding author upon reasonable request.
